# Awareness and openness to the use of PrEP among a nationally representative sample of South African adults

**DOI:** 10.11604/pamj.2024.48.95.34137

**Published:** 2024-07-09

**Authors:** Lungile Nkosi, Queen Dooshima Mmem, Tina Ngufan Tsafa, Joy Ngodoo Gwar, Israel Terungwa Agaku

**Affiliations:** 1 Chisquares Inc. Atlanta, Georgia, United States of America,; 2 School of Health Systems and Public Health, University of Pretoria, Pretoria, South Africa,; 3 Department of Mass Communication, Benue State University, Makurdi, Nigeria,; 4 Federal Medical Centre, Makurdi, Benue State, Nigeria

**Keywords:** PrEP, South Africa, HIV, openness, awareness, adults

## Abstract

**Introduction:**

South Africa adopted for pre-exposure prophylaxis (PrEP) in 2016, becoming the first African country to do so. Yet to date, uptake has been underwhelming, only about 165,000 South Africans were reported to be on PrEP in mid-2021. Lack of awareness has been cited as a contributory factor for the low uptake, but this has never been examined using a nationally representative sample.

**Methods:**

we investigated this among a national sample of HIV seronegative adults. Data were from the 2017/2018 South African National HIV Prevalence, Incidence, Behaviour and Communication Survey. Awareness and openness to using PrEP were self-reported. Weighted percentages were calculated overall and by demographic characteristics.

**Results:**

overall, only 3.2% of seronegative adults spontaneously reported PrEP as a way of preventing HIV. Overall, 69.6% were open to using PrEP, from 58.2% in Western Cape, to 78.5% Northern Cape. Openness was highest among the youngest age group (18-29 years, 78.3%) and lowest among the oldest (60+ years, 45.6%). Striking racial differences were observed with openness among Black Africans (75.4%) being 2.5 times higher than Whites (29.0%). Among women, openness was 64.7% among those currently pregnant, 80.4% among those pregnant in the past two years but not now, and 67.8% among those who were not pregnant in the past two years (χ(2)=134.2, p<0.001). Among males, openness was higher among those circumcised (75.6%) than uncircumcised (64.5%).

**Conclusion:**

planning for broad-scale implementation of PrEP within the South African context could build on knowledge gained from recent implementation and scale-up of relevant biomedical interventions (e.g. ART, voluntary medical male circumcision, and family planning).

## Introduction

South Africa bears the largest share of the global HIV epidemic, with an estimated 7.9 million people of all ages living with HIV in 2017 [1], 4.4 million of whom are on antiretroviral treatment (ART) [2]. Test and treat a strategy that recommends screening for HIV infection among populations at risk, and early treatment for those infected by HIV was introduced in South Africa in 2016, yet there were approximately 231,000 new HIV infections in 2017 [2,3]. Prevalence is higher among men who have sex with men (MSM), transgender women, sex workers, and people who inject drugs [2,4]. Reasons for increased rates of HIV in South Africa can be tied to several social, economic, gender, and health-based factors that have likely worsened with the recent COVID-19 pandemic [5]. Recent gains have been made in reducing HIV deaths and curtailing new infections but for these to be sustained, there needs to be rapid uptake of new prevention and risk-reduction interventions, including PrEP [6].

In 2015, the World Health Organization (WHO) recommended daily oral PrEP as a prevention strategy for people at “substantial risk” of HIV infection [3]. PrEP is defined by the WHO as “the use of antiretroviral drugs by HIV-negative people who are at substantial risk of acquiring HIV before potential exposure to HIV to prevent HIV acquisition” [3]. The appeal of PrEP is that it does not require the cooperation of one's sexual partner, unlike certain other HIV prevention options such as male and female condoms, verifying a partner's HIV status, or ensuring a partner living with HIV is virally undetectable. Prevailing gender norms may hinder females from implementing such traditional prevention measures, putting them at high risk for HIV transmission [7,8].

In addition, PrEP users reported that they were able to engage in sexual risk behaviours regardless of the known risk of HIV infection without compromising the perceived benefits from condomless sex such as sexual satisfaction [9]; PrEP also gave them a sense of return to normalcy as they were able to plan for pregnancy [10]. South Africa adopted PrEP in 2016, becoming the first African country to do so [11]. The current preferred regimen in South Africa is oral Tenofovir/emtricitabine (TDF/FTC) as a fixed-dose combination [12]. Indicated populations in South Africa include adolescent girls and young women, MSM, people who have more than one sexual partner, people who inject drugs, people with a recent history of sexually transmitted infections, people who recognize their own risk and request PrEP, serodiscordant couples if the HIV partner is not virally suppressed, and sex workers. Also indicated are individuals whose partners have unknown HIV status, a history of inconsistent or no condom use, or having sex whilst under the influence of alcohol or recreational drugs [12,13]. To ensure that supply meets demand, South Africa has accessed generic PrEP formulations, thereby reducing medication price, and has integrated it with other health services [14].

The South African PrEP model is based on an equity approach that prioritized vulnerable groups such as sex workers who have the highest HIV prevalence in South Africa and face levels of stigma and discrimination [14]. This demographic was the first group to be offered PrEP in 2016. The phased PrEP rollout was then expanded in 2017 to facilities providing services to MSM, and then to students at selected university campus clinics, followed by primary health care facilities [15]. Recently, since 2018, access to PrEP in South Africa has expanded and the medication is currently available in public sector health facilities within the country in addition to demonstration projects and observational studies [16-18].

However, uptake of PrEP remains relatively low, with only about 165,000 South Africans reported to be on PrEP as of April 2021, a stark contrast to the high HIV prevalence rate in the country [19]. Among the suggested drivers of low uptake is a lack of awareness about the availability of the drug as well as the stigma around the use of PrEP [6,20]. Several small studies have been conducted to explore awareness and utilization of PrEP in South Africa, but to date, no nationally representative study has been done. To fill this knowledge gap, this study assessed awareness and openness to using PrEP among a nationally representative sample of South African adults. Because of the novelty of PrEP at the time of the study in South Africa (a high HIV prevalence setting), we explored the study endpoints in the entire population of HIV seronegative individuals, not only those for whom PrEP is indicated.

## Methods

**Study design, setting, and participants:** we analysed the fifth wave of the South African National HIV Prevalence, HIV Incidence, Behaviour and Communication Survey (SABSSM V) [21], a nationally representative, face-to-face, household survey of the South African civilian population. Individuals of all ages living in households (including hostels) were eligible to participate. The response rate was 82.2%. The survey employed a multi-stage stratified random cluster sampling approach [1], with 15 visiting points (households) systematically selected from a sampling frame consisting of a sub-sample of 1,000 small area layers (SALs) drawn from the 2015 national population sampling frame developed by Statistics South Africa (StatsSA). The StatsSA sampling frame consisted of 84,907 SALs. For a representative sample, disproportionate stratification of SALs was done by race group, province, and geographic/locality type (i.e. urban, rural informal [tribal area], and rural formal [farms]) [1]. The survey included both an interview and a laboratory component to gather comprehensive data.

### Variables and data sources/measurement

***HIV status and HIV perceived risk:*** HIV status was assessed in both the interview (self-reported HIV status) and laboratory (objectively ascertained HIV status through a dried blood spot sample collected by finger-prick, or by heel prick in infants) components of the survey [1]. Among HIV negative individuals or those with unknown status, the survey asked, “On a scale of 1 to 4 (with 1 being low and 4 being high), how would you rate yourself in terms of risk of becoming infected with HIV?” Scores of ≥3 were classified as high perceived risk. Data were also collected on lifetime and past-year sexual activity, including the number of partners, type of partners, route of sex, condom use frequency, and other demographic characteristics.

***Awareness and interest in PrEP:*** unaided recall, also known as spontaneous recall, was used to evaluate how well participants could remember PrEP as one of many HIV prevention methods without the help of any external cues or clues. This was in response to the question “Can you tell me all the ways you know how to prevent HIV infection?” In the script provided to the survey administrators fielding the survey using face-to-face interviews, they were instructed: “DO NOT READ OUT OPTIONS, MULTIPLE RESPONSES POSSIBLE”. The responses captured were therefore spontaneous and based on the respondent's recall. The survey administrator could check off the respondent's answer with the closest of the provided options, or if not one of the options provided, could specify it under 'Other'. Following this question assessing awareness of PrEP, openness to using PrEP was assessed thus: “Scientists are now studying a medication where, if taken orally every day, can reduce a person's chances of getting HIV infection. If such a medication was available, would you want to take it?” Categorical response options were “Yes”, “No”, or “Don't know”. An answer of “Yes” was classified as being open to using PrEP, whereas those who answered “No”, or “Don't know” were classified as not being open to using PrEP.

***Other clinical, behavioural, and demographic characteristics:*** data were collected on ever and past-year sexual activity, circumcision (for males), binge drinking (having ≥5 drinks for males or ≥4 drinks for females on the same occasion during the past month), and ever recreational use of substances of abuse. Substances assessed in the survey included marijuana, cocaine, amphetamines, inhalants, hallucinogens, opiates, and others not otherwise specified. Regarding the route of substance use, participants were also asked about any history of injection drug use. Demographic characteristics include gender, province, sexual orientation, race, marital, and nativity status.

**Bias:** the study aimed to minimize bias by employing a multi-stage stratified random cluster sampling approach and weighting the data to yield nationally representative estimates. The population was divided into strata based on key characteristics, and random clusters were selected within each stratum. Data were collected by trained field workers through one-on-one interviews. Each response was anonymously linked to a unique barcode, which was scanned onto an electronic questionnaire and attached to a blood specimen. Informed consent was provided by all adult respondents.

**Quantitative variables:** key quantitative variables included perceived risk of HIV infection (measured on a scale of 1 to 4), awareness of PrEP (measured through unaided recall), and openness to using PrEP (categorical responses of “Yes,” “No,” or “Don't know”).

**Statistical methods:** data were weighted to yield nationally representative estimates. Among HIV seronegative adults, we estimated the percentage who reported awareness of PrEP based on unaided recall, as well as the percentage open to using PrEP. Percentages were compared with two-sided Chi-squared tests at p < 0.05. Adjusted prevalence ratios were calculated in an exploratory Poisson regression model to determine factors associated with openness towards daily oral PrEP among all HIV seronegative individuals. Blinder Oaxaca decomposition analysis was performed to see what factors explained the gap in PrEP openness between past-year sexually active seronegative individuals with high vs low perceived HIV risk. Analyses were performed in Stata Version 14.0

**Ethics approval:** this study was performed in line with the principles of the Declaration of Helsinki. This study was deemed non-human subject research as all data were publicly available, de-identified, secondary datasets. Ethical approval was therefore not sought.

## Results

**Participants:** a total of 13,610 participants were analysed in this study. The demographic characteristics of the participants were diverse, representing various age groups, genders, and geographic locations (Table 1).

**Table 1:** percentage of HIV seronegative adults who reported awareness of PrEP as an HIV prevention measure based on unaided recall, and the percentage open to using PrEP, South African National HIV, Behaviour, and Health Survey, 2017/2018

Characteristic	N	Spontaneous recall of PrEP % (95%CI)	Open to using daily oral PrEP when described % (95%CI)
Total	13,610	3.2 (2.7-3.6)	69.6 (68.2-71.0)
**Province**			
Western Cape	1,308	4.3 (3.1-5.5)	58.2 (54.9-61.6)
Eastern Cape	1,116	5.1 (3.4-6.9)	63.9 (60.1-67.7)
Northern Cape	859	0.8 (0.2-1.4)	78.5 (75.2-81.8)
Free State	655	11.8 (8.5-15.0)	79.5 (75.6-83.3)
KwaZulu-Natal	4,023	2.6 (1.5-3.7)	67.8 (64.8-70.8)
North-West	920	1.0 (0.3-1.8)	76.3 (72.6-80.0)
Gauteng	2,229	1.4 (0.6-2.3)	73.9 (70.7-77.1)
Mpumalanga	1,456	3.3 (1.7-4.9)	76.3 (72.5-80.1)
Limpopo	1,044	2.3 (1.0-3.7)	67.9 (63.9-71.9)
**Geographic location**			
Urban	7,555	3.2 (2.6-3.8)	69.2 (67.4-70.9)
Rural informal	4,540	2.9 (2.0-3.7)	71.8 (69.5-74.1)
Rural (farms)	1,515	4.2 (2.6-5.8)	65.4 (60.9-69.9)
**Age, years**			
18 to 29	5,027	3.4 (2.6-4.2)	78.3 (76.3-80.4)
30 to 39	2,489	2.9 (1.9-3.9)	76.8 (73.9-79.7)
40 to 49	1,710	4.8 (3.2-6.5)	65.4 (61.4-69.3)
50 to 59	1,944	2.1 (1.2-3.1)	62.1 (58.5-65.7)
60+	2,440	2.5 (1.6-3.4)	45.6 (42.1-49.0)
**Sexual orientation ^a^**			
Heterosexual males/sexually inactive males	4,610	3.3 (2.5-4.1)	70.9 (68.7-73.1)
Heterosexual females/sexually inactive females	7,171	3.2 (2.6-3.8)	69.3 (67.5-71.1)
Homosexual males	903	3.2 (1.2-5.2)	65.7 (59.0-72.3)
Lesbians	916	1.9 (0.5-3.2)	62.8 (55.2-70.4)
**Race**			
Black African	10,141	3.0 (2.5-3.5)	75.4 (73.9-76.9)
White	704	2.9 (1.5-4.3)	29.0 (24.3-33.6)
Coloured	1,900	4.7 (3.4-6.0)	69.5 (66.6-72.3)
Indian/Asian	840	2.9 (0.1-6.0)	49.1 (41.6-56.6)

Note: PrEP = pre-exposure prophylaxis. Subgroup sample sizes may not add up to the total because of missing or indeterminate responses. ^a^Derived variable generated using identified sex of respondent and gender of their reported sexual partners.

### Main Results

***Awareness of PrEP and openness to using it among all seronegative adults:*** unaided recall of PrEP as an HIV preventive measure was low; only 3.2% of seronegative adults spontaneously reported PrEP as a way of preventing HIV, with no significant differences seen across subgroups, except by province. Openness was lowest in Northern Cape (0.8%), and highest in Free State (11.8%) (χ(8)=454.6, p<0.001) (Table 1).

Of all seronegative adults, 69.6% were open to using PrEP, with wide geographic variability observed across South Africa's nine provinces (from 58.2% in Western Cape, to 79.5% in Free State, χ(8)=457.6, p<0.001). Openness was highest among the youngest age group (18-29 years, 78.3%) and lowest among the oldest (60+ years, 45.6%). Striking racial differences were observed with openness to PrEP by Black Africans (75.4%) being 2.5 times higher than Whites (29.0%) (χ(3)=2125.9, p<0.001). By education, those still in post-secondary school education had the highest openness (79.6%), whereas openness was lowest at both extremes of education, i.e., those with no schooling (54.9%) and those with the highest schooling (54.4%) (Table 1.1). Pensioners reported the lowest openness to PrEP (56.6%), whereas the highest openness was seen among those with no monthly income (χ(5)=403.7, p<0.001). Gender difference in PrEP openness was not statistically significant (70.3% vs 69.0%, χ(1)=4.4, p=0.3625). Those who have never been married reported the highest openness (76.0%), compared to those married (62.5%), or divorced/widowed/separated (55.1%) (χ(2)=659.2, p<0.001). Current living arrangements were also associated with PrEP openness; openness was highest among those reporting they were in a steady unmarried relationship but not living together (77.0%) and lowest among those married and living together with their spouse (61.5%) (χ(4)=375.4, p<0.001). Openness was significantly lower among those with a disability than with none (54.6% vs 70.2%) as well as those with comorbidity than with none (61.3% vs 72.1%) (all p<0.05). While binge drinkers were more open to PrEP than those not reporting binge drinking (76.1% vs 68.5%), those reporting injection drug use were less open to PrEP than those not reporting injection drug use (53.2% vs 69.8%) (all p<0.05). Individuals who had ever cared for a child with AIDS also reported higher openness towards PrEP than those who had never (73.6% vs 69.3%).

**Table 1.1:** percentage of HIV seronegative adults who reported awareness of PrEP as an HIV prevention measure based on unaided recall, and the percentage open to using PrEP, South African National HIV, Behaviour, and Health Survey, 2017/2018

Education			
No school	1,325	1.7 (0.6-2.8)	54.9 (50.0-59.7)
≤ 6^th^ grade	1,600	1.9 (0.9-2.8)	62.8 (58.9-66.7)
7^th^ to 12^th^ grade	7,026	3.5 (2.8-4.1)	72.3 (70.5-74.1)
Further studies incomplete	261	5.7 (1.6-9.9)	79.6 (71.7-87.6)
Further studies completed	873	4.3 (2.2-6.3)	54.4 (49.2-59.6)
**Past-month Income source**			
Salary/earnings	3,650	3.5 (2.6-4.5)	68.4 (65.9-70.9)
Contributions by family members or relatives	408	5.8 (2.5-9.0)	69.4 (62.2-76.6)
Government pensions/grants	1,934	2.5 (1.5-3.5)	56.6 (52.7-60.6)
Grants/donations by private welfare organizations	764	2.6 (1.0-4.3)	65.3 (60.0-70.7)
Other	459	3.6 (0.9-6.4)	60.9 (53.5-68.3)
No monthly income	5,578	3.3 (2.6-4.0)	75.4 (73.4-77.3)
**Nationality**			
South African	12,429	3.4 (2.9-3.9)	69.9 (68.5-71.3)
Documented migrant	253	3.5 (0.7-7.7)	68.8 (59.9-77.7)
Undocumented migrant/asylum seeker/refugee/other	94	1.0 (1.0-3.0)	53.3 (37.2-69.4)
**Sex**			
Man	5,513	3.3 (2.5-4.0)	70.3 (68.2-72.4)
Woman	8,087	3.1 (2.5-3.7)	69.0 (67.2-70.8)
**Marital status**			
Married	3,881	3.2 (2.4-4.1)	62.5 (59.9-65.0)
Never Married	7,469	3.5 (2.8-4.2)	76.0 (74.3-77.7)
Divorced/separated/widowed	1,451	2.9 (1.5-4.3)	55.1 (50.9-59.3)
**Living arrangements**			
Married, living with husband/wife	3,490	3.2 (2.4-4.0)	61.5 (58.8-64.2)
Married, living apart	1,835	2.8 (1.4-4.3)	74.3 (70.8-77.8)
Living together with boyfriend/girlfriend/civil union	860	3.0 (1.2-4.8)	73.8 (68.9-78.8)
In a steady relationship but not living together	2,602	2.9 (1.9-3.9)	77.0 (74.3-79.8)
Single; not in a steady relationship	3,950	4.1 (3.1-5.1)	69.4 (66.9-71.9)
**Disability**			
Yes	511	2.4 (0.6-4.3)	54.6 (47.0-62.2)
No	12,275	3.4 (2.9-3.9)	70.2 (68.8-71.6)
**Non-HIV comorbidity ^a^**			
Yes	10,244	3.1 (2.2-3.9)	61.3 (58.6-64.1)
No	3,366	3.2 (2.6-3.7)	72.1 (70.5-73.7)

Note: PrEP= pre-exposure prophylaxis. Subgroup sample sizes may not add up to the total because of missing or indeterminate responses. ^a^ Non-HIV comorbidities assessed were hypertension, diabetes, tuberculosis, cancer, and heart disease, all of which were self-reported.

Among women, openness was 64.7% among those currently pregnant, 80.4% among those pregnant in the past two years but not now, and 67.8% among those who were not pregnant in the past two years (χ(2)=134.2, p<0.001). Among males, openness was higher among those circumcised (75.6%) than uncircumcised (64.5%) (χ(1)=249.2, p<0.001). For both males and females combined, those sexually active in the past year reported significantly higher openness than those not active (74.1% vs 62.6%, χ(1)=322.3, p<0.001). Of those engaging in sexual activity in the past year, openness varied by type of sexual partner, sex route, and number of sexual partners. By type of partner reported (non-mutually exclusive), openness was: with spouse (65.0%), steady unmarried partner (73.5%), girl/boy-friend (81.9%), other casual sex partners (77.6%), and with a commercial sex worker (75.7%). By route of sex reported (non-mutually exclusive), openness was vaginal (74.4%), anal (82.2%), and oral (80.5%). Of those sexually active in the past year, openness to PrEP was higher among those with regular condom use than not (82.6% vs 71.1%). Those who got money for sex reported higher PrEP openness (87.8%) than those who gave money for sex (75.6%).

### Other analyses

***Multivariable analysis of factors associated with openness towards PrEP use among all HIV seronegative individuals:*** within multivariable analysis, the likelihood of being open towards PrEP was significantly higher among adults in Northern Cape (APR=1.19), Free State (APR=1.28), Northwest (APR=1.10), Gauteng (APR=1.14), and Mpumalanga provinces (APR= 1.14), when compared to Western Cape. Whites (APR=0.44) and Indians/Asians (APR=0.68) were less likely to be open towards PrEP compared to Black Africans (Table 2). Compared to those with no formal schooling, likelihood of being open to PrEP was higher among those with 7^th^ to 12^th^ grade education (APR= 1.14) and some post-secondary education but not yet completed (APR=1.19). Adults whose income came from Grants/donations by private welfare organizations were more likely to be open towards PrEP compared to those with no monthly income (APR=1.12). Compared to young adults aged 18-29 years, the likelihood of being open towards PrEP was lower among those aged 40-49 years (APR=0.90), 50-59 (APR=0.88), and 60+ years (APR=0.67). Furthermore, those who were never sexually active were less likely to be open to taking PrEP compared to those reporting sexual activity within the past year (APR=0.85) (all p<0.05) (Table 2.1).

**Table 2:** adjusted prevalence ratios for factors associated with openness towards using daily oral PrEP among HIV seronegative adults (N=13, 610)

Characteristics	Prevalence ratios with 95% confidence intervals	P-value
**Province**		
Western Cape	1.00 (reference category)	
Eastern Cape	1.01 (0.92-1.11)	0.861
Northern Cape	1.19 (1.10-1.29)	<0.001
Free State	1.28 (1.17-1.41)	<0.001
KwaZulu-Natal	1.09 (0.99-1.19)	0.08
North-West	1.10 (1.01-1.21)	0.038
Gauteng	1.14 (1.04-1.25)	0.004
Mpumalanga	1.14 (1.03-1.25)	0.008
Limpopo	1.07 (0.96-1.18)	0.216
**Age, years**		
18 to 29	1.00 (reference category)	
30 to 39	0.99 (0.93-1.04)	0.616
40 to 49	0.90 (0.84-0.97)	0.005
50 to 59	0.88 (0.82-0.95)	0.001
60+	0.67 (0.61-0.75)	<0.001
**Sex**		
Man	1.00 (reference category)	
Woman	1.01 (0.97-1.06)	0.687
Race		
Black African	1.00 (reference category)	
White	0.44 (0.37-0.53)	<0.001
Coloured	1.02 (0.95-1.09)	0.548
Indian/Asian	0.68 (0.58-0.79)	<0.001
**Highest educational attainment**		
No school	1.00 (reference category)	
≤ 6^th^ grade	1.06 (0.95-1.18)	0.284
7^th^ to 12^th^ grade	1.14 (1.04-1.25)	0.005
Further studies incomplete	1.19 (1.04-1.36)	0.012
Further studies completed	1.07 (0.95-1.21)	0.283
Don't know	1.06 (0.63-1.79)	0.828
**Monthly income, ZAR**		
No monthly income	1.00 (reference category)	
Salary/earnings	0.98 (0.94-1.03)	0.533
Contributions by family members or relatives	0.96 (0.85-1.08)	0.517
Government pensions/grants	1.00 (0.91-1.09)	0.940
Grants/donations by private welfare organizations	1.12 (1.03-1.23)	0.012
Other	0.94 (0.84-1.06)	0.348

Note: Adjusted prevalence ratios were calculated in a multivariable Poisson regression model that adjusted for all factors listed in table.

**Table 2.1:** adjusted prevalence ratios for factors associated with openness towards using daily oral PrEP among HIV seronegative adults (N=13, 610)

Any substance use behaviour		
Nonuser	1.00 (reference category)	
User	1.02 (0.95-1.10)	0.530
**Living arrangements**		
Married, living with husband/wife	1.00 (reference category)	
Married, living apart	1.03 (0.96-1.10)	0.424
Living together with boyfriend/girlfriend/civil union	0.99 (0.91-1.07)	0.762
In a steady relationship but not living together	1.04 (0.98-1.11)	0.192
Single; not in a steady relationship	0.96 (0.90-1.03)	0.242
**Last sexual activity**		
Within the past year	1.00 (reference category)	
Over a year ago	0.97 (0.92-1.03)	0.338
Don't know	0.96 (0.82-1.14)	0.651
Never sexually active	0.85 (0.77-0.94)	0.002

Note: Adjusted prevalence ratios were calculated in a multivariable Poisson regression model that adjusted for all factors listed in table.

***Explaining the gap in PrEP openness between past-year sexually active seronegative individuals with high vs low perceived HIV risk:*** of those sexually active in the past year, 81.2% of those perceiving their HIV risk as high were open to PrEP, vs 72.4% of those perceiving their HIV risk as low (χ2(1)=29.0, p<0.001), a gap of 8.8 percentage points. Within decomposition analysis, demographic factors (particularly race and age) played a more important role as explanatory variables than sex behaviours (Table 3). In total, 60.1% of the gap in PrEP openness between those with perceived high vs low HIV risk was attributable to differences in the following set of demographic variables combined: age, gender, urbanicity, race, and education. By individual demographic variables, differences in racial distribution alone explained 49.6% of the total gap in PrEP openness between those perceiving their HIV risk as high vs low; age differences explained 21.3% of the gap, differences in urbanicity explained 8.6% of the gap, while educational differences explained 8.3%. Gender differences did not explain to a significant extent any of the observed gaps in PrEP openness between those with high vs low perceived HIV risk.

**Table 3:** Blinder-Oaxaca decomposition analysis of factors explaining the gap in PrEP openness between sexually active persons perceiving their HIV risk as high (n=1423) vs low (n=5309), South African National HIV, Behaviour, and Health Survey, 2017/2018

Construct	Variables	Percentage of gap explained % (95%CI)	P-value
Substance use	Ever alcohol use, binge drinking, ever use of illicit drugs	6.1 (2.4-9.8)	<0.001
Route of sex	Anal, oral, vaginal	5.6 (2.6-8.6)	<0.001
Type of sex partner	Spouse, partner, casual sex partner, commercial sex worker	26.6 (12.2-41.0)	<0.001
Race	Black African vs other	49.6 (35.4-63.7)	<0.001
Age	Continuous age	21.3 (12.7-30.0)	<0.001
Education	highest education attained	8.3 (3.9-12.6)	<0.001
Urbanicity	metro	8.6 (3.5-13.6)	0.001
All demographics	Age, gender, urbanicity, race, education	60.1 (42.8-77.3)	<0.001

Note: PrEP = pre-exposure prophylaxis

In terms of sexual activity and perceived risk, the types of relationships within which people were having sex (with a spouse, unmarried partner, casual sex partner, or commercial sex worker), was a more important determinant of PrEP openness, than any of the following: the number of sexual partners they reported, the route of sex (anal, oral, or vaginal, together), or their condom use. Of the gap in PrEP openness between those perceiving high vs low HIV risk, 26.6% was explained by the type of sexual relationship, 5.6% was explained by the sex route, while neither the number of sexual partners (p=0.711) nor condom use (p=0.202) explained to a significant extent any of the observed gap in PrEP openness between those with high vs low perceived HIV risk. Other high-risk behaviours of a non-sexual nature (e.g., binge drinking, injection drug use, other substance abuse behaviours), explained 6.1% of the observed gap in PrEP openness between those perceiving high vs low HIV risk.

## Discussion

Unaided recall for PrEP as an HIV prevention method was very low, suggesting ever use must be similarly low. Previous studies examining ever use of PrEP in South Africa have shown low use [6,22,23], including a recent study by Lanham *et al*. [24] that revealed that adolescent girls and young women were largely unaware of PrEP. Nonetheless, the high level of interest in PrEP found in our study shows opportunities to scale the intervention, especially among subgroups with a high level of interest. Within the multivariable analysis, we found that certain provinces with higher HIV prevalence also had higher openness towards PrEP use, which is important for targeted interventions as it would be logistically easier to target delivery of PrEP by a well-defined geographic area than by some other individual-level characteristic as the indicated individuals may be dispersed in the population and may not be willing to come forth, especially if the characteristic is associated with stigma. Other observed differences in openness to PrEP along the lines of race, age, income source, and sexual behaviours may be attributable to differences in perceived risk for HIV [25,26].

A holistic or person-centered approach rather than a disease-specific approach will benefit public health when promoting or scaling the use of PrEP in South Africa [27,28]. PrEP may indeed eliminate the risk of HIV if properly and consistently taken but risks of contracting other sexually transmitted infections may remain, as well as the risk of unwanted pregnancies [29,30]. Framing of public health messages should therefore emphasize that being on PrEP does not warrant condomless sex. Adherence support may also be needed as the daily oral dosing required for PrEP may lead to suboptimal adherence from factors such as pill fatigue, side effects, confidentiality challenges, and stigma, similar to what has been observed for daily oral ART [31,32]. Two dimensions of stigma have been identified as barriers to PrEP uptake use, especially among females: fear of being labelled as HIV positive, and fear of being labelled as sexually 'promiscuous' or as a sex worker [24]. Consequently, some women have expressed worries about parents or partners discovering the tablets or hearing them rattle in the bottle [24]. To reduce stigma, there is a need for social media and population-level campaigns that aim to normalize PrEP use and remove perceived stigma [33].

HIV, socioeconomic gaps, and gender equality are inextricably linked in the South African context and have fuelled in no small measure the disproportionately higher HIV prevalence among women [7]. Women seeking temporary relief, shelter, and amenable living conditions in acutely insecure contexts become potential targets for exploitation and human trafficking [5]. As of 2020, 53% of all people living with HIV were women and girls [5]. Notably, we did not see a significant gender difference in PrEP openness. PrEP however holds the potential to increase female empowerment in the region, based on findings from past studies [10,34]. Eakle *et al*. [35] carried out a study to explore the acceptability of oral PrEP prior to implementation among female sex workers in South Africa; overall, there was strong acceptability of PrEP among participants and positive anticipation for the imminent delivery of PrEP in the local sex worker clinics. Themes arising from the discussions exploring aspects of PrEP acceptability included awareness and understanding of PrEP, PrEP motivations including choice, control, and vulnerability, managing PrEP risks and worries, and destigmatizing and empowering PrEP delivery. Effective integration of PrEP into existing services should also include comprehensive health programming where ART is also available, appropriate messaging, and support [30,35].

### Limitations

Firstly, the cross-sectional data capture only one single snapshot in time; the PrEP landscape in South Africa may have evolved significantly since these data were collected in 2017/2018. Secondly, intentions may not necessarily translate into behaviours as far as using PrEP; acceptability, availability, and affordability may all play a part as well [36]. Social norms regarding PrEP use may also influence individual behaviour [37,38]. Thirdly, self-reported information may be subject to misclassification and several other social and cognitive biases. Fourthly, as this study aimed to examine awareness of a relatively new medical innovation and explored this in a hypothetical context in the survey, we examined the endpoints in the entire population of HIV seronegative individuals, not only those for whom PrEP is indicated. Finally, this study only assessed daily oral PrEP dosing and did not capture openness towards newer modalities of PrEP intake such as PrEP on demand (or PrEP 2-1-1), as well as longer-acting regimens (such as injectable regimens). Event-based oral dosing requires 2 pills between two and 24 hours before each sexual event, plus one pill 24 hour after the first two pills, and the last pill 48 hours after the first two pills (i.e. 4 pills combined per event, every event) [39,40]. Intramuscular PrEP injections on the buttocks administered once every two months at a doctor's office (i.e., 6 injections a year for year-round prevention) were approved by the FDA in December 2021 but are likely not available in South Africa [41].

## Conclusion

Awareness of PrEP was low; however, close to 7 in 10 seronegative adults were open to taking PrEP if it were available. Planning for broad-scale implementation of PrEP within the South African context could build on knowledge gained from recent implementation and scale-up of relevant biomedical interventions (e.g., ART, voluntary medical male circumcision, and family planning). Efforts to promote PrEP uptake should focus on achieving a positive equity impact and reduce existing disparities. As such, groups at highest risk for HIV transmission should be targeted, particularly adolescent girls and young women.

### What is known about this topic

- *South Africa became the first African country to formally adopt and implement PrEP in 2016, but the uptake of PrEP remains low.*

### What this study adds

- *Awareness of PrEP was low; however, close to 7 in 10 seronegative adults were open to taking PrEP if it were available.*
